# Communication about HIV and death: Maternal reports of primary school-aged children's questions after maternal HIV disclosure in rural South Africa

**DOI:** 10.1016/j.socscimed.2016.10.031

**Published:** 2017-01

**Authors:** Tamsen J. Rochat, Joanie Mitchell, Anina M. Lubbe, Alan Stein, Mark Tomlinson, Ruth M. Bland

**Affiliations:** a Africa Health Research Institute, University of KwaZulu-Natal, South Africa; b Human and Social Development Research Programme, Human Sciences Research Council, South Africa; c MRC/Developmental Pathways to Health Research Unit, School of Clinical Medicine, Faculty of Health Sciences, University of Witwatersrand, South Africa; d Section of Child and Adolescent Psychiatry, Department of Psychiatry, University of Oxford, United Kingdom; e Department of Psychology, Stellenbosch University, Stellenbosch, South Africa; f MRC/Wits Rural Public Health and Health Transitions Research Unit (Agincourt), School of Public Health, Faculty of Health Sciences, University of the Witwatersrand, South Africa; g School of Public Health, Faculty of Health Sciences, University of Witwatersrand, Johannesburg, South Africa; h Royal Hospital for Sick Children, Institute of Health and Wellbeing, University of Glasgow, Glasgow, United Kingdom

**Keywords:** Children, HIV disclosure, Death, Questions

## Abstract

**Introduction:**

Children's understanding of HIV and death in epidemic regions is under-researched. We investigated children's death-related questions post maternal HIV-disclosure. Secondary aims examined characteristics associated with death-related questions and consequences for children's mental health.

**Methods:**

HIV-infected mothers (*N* = 281) were supported to disclose their HIV status to their children (6–10 years) in an uncontrolled pre-post intervention evaluation. Children's questions post-disclosure were collected by maternal report, 1–2 weeks post-disclosure. 61/281 children asked 88 death-related questions, which were analysed qualitatively. Logistic regression analyses examined characteristics associated with death-related questions. Using the parent-report Child Behaviour Checklist (CBCL), linear regression analysis examined differences in total CBCL problems by group, controlling for baseline.

**Results:**

Children's questions were grouped into three themes: ‘*threats’*; *‘implications’* and *‘clarifications’*. Children were most concerned about the threat of death, mother's survival, and prior family deaths. In multivariate analysis variables significantly associated with asking death-related questions included an absence of regular remittance to the mother (*AOR* 0.25 [CI 0.10, 0.59] *p* = 0.002), mother reporting the child's initial reaction to disclosure being “frightened” (*AOR* 6.57 [*CI* 2.75, 15.70] *p*=<0.001) and level of disclosure (full/partial) to the child (*AOR* 2.55 [*CI* 1.28, 5.06] *p* = 0.008). Controlling for significant variables and baseline, all children showed improvements on the CBCL post-intervention; with no significant differences on total problems scores post-intervention (β -0.096 *SE*1.366 *t* = -0.07 *p* = 0.944).

**Discussion:**

The content of questions children asked following disclosure indicate some understanding of HIV and, for almost a third of children, its potential consequence for parental death. Level of maternal disclosure and stability of financial support to the family may facilitate or inhibit discussions about death post-disclosure. Communication about death did not have immediate negative consequences on child behaviour according to maternal report.

**Conclusion:**

In sub-Saharan Africa, given exposure to death at young ages, meeting children's informational needs could increase their resilience.

## Highlights

• Children living in HIV epidemic communities are exposed to death at young ages.
• Primary-school aged children understand information on HIV, illness and death.
• Intervention training successfully prepared mothers to communicate about death.
• Communication about death did not increase children’s mental health problems.
• HIV disclosure creates opportunities to communicate with and reassure children.

## 1 Introduction

Many children in Africa are at risk of parental bereavement, either as a direct result of their parents’ HIV infection or indirectly through HIV-related social and health adversities (Hosegood, 2009). The effects of HIV on children are heightened in endemic regions, including South Africa, where the antenatal HIV prevalence is as high as 40%, and approximately half of all households include an adult on HIV treatment (Bor et al., 2011). Children are exposed to the effects of HIV at a family and community-level from an early age (Rochat et al., 2011). HIV-infected parents face challenges adjusting to parenting with HIV, including preparing their children for periods of illness and ultimately their possible death, both of which have negative psychological impacts for children (Sherr et al., 2014).

Young children (4–6 years) are able to develop an understanding of death using a ‘naïve theory of biology’ whereby death is understood as having a biological cause (Vlok and de Witt, 2012). During childhood, these naive theories develop, leading to a mature understanding of death that incorporates mastery of several concepts including: inevitability (that living things must die eventually); universality (that inevitability applies to all living things); irreversibility (recognition that the dead cannot return); cessation (that death is characterised by bodily processes ceasing to function); and causation (that death is caused by breakdown in bodily function) (Slaughter, 2005). While traditional psychoanalytic and child developmental theories (particularly Piagetian) suggest that mastery of a mature concept of death emerges in later childhood (9–11 years), more recent intuitive theories argue that children's experience (Hunter and Smith, 2008) and exposure to biological information frame the timing and order in which children master concepts of death (Slaughter and Griffiths, 2007). Providing children with developmentally appropriate information mitigates some of the effects of parental illness and death on the child (Dunning, 2006).

In high-income settings, researchers have shown that primary-school-aged children have few preconceived ideas about the meaning of HIV (Kennedy et al., 2010) and a limited developmental capacity to understand HIV and its potential to cause parental death (Murphy et al., 2006). However, this may not be generalizable to HIV-endemic low-income settings (Rochat et al., 2014). South African research has shown that children have at least a naïve understanding of human disease processes, and in the context of high exposure to death assimilate experience and understanding of external and internal causes of death (Vlok and de Witt, 2012). It is also clear that children's understanding of death is developmentally framed (Christ and Christ, 2006) but strongly influenced by previous exposures (Hunter and Smith, 2008).

In epidemic regions, where approximately a third of children live with an HIV-infected parent, most frequently a mother (Short and Goldberg, 2015) parental support to deal with parent-child communication about HIV and death is an important public health priority. Maternal HIV disclosure has generally been found to be beneficial for children, parents and families (Qiao et al., 2013) and became a World Health Organization recommendation in 2012 (WHO, 2011). However, there is a dearth of interventions to support parents (Kennedy et al., 2015), in particular those with younger children, with this challenging task.

The Amagugu disclosure intervention (Rochat et al., 2013) addressed these parental needs. The conceptual framework of the intervention is described in open access format elsewhere (Rochat et al., 2016) and includes a focus on increasing parent-child communication about HIV (generally, and through parental HIV disclosure) and assisting mothers to prepare for periods of illness. Mothers were encouraged to make either full (using the words ‘HIV’) or partial (using the word ‘virus’) disclosures (Qiao et al., 2013). The training addressed maternal preparation for children's emotional reactions and questions following disclosure, including questions about parental death (see supplementary material).

The intervention led to high rates of disclosure (Rochat et al., 2014), including full disclosure (61%). Maternal reports of children's questions post-disclosure illustrated that children had some understanding of HIV illness, transmission and treatment, and that disclosure raised discussion about the possibility of maternal illness and death amongst some children. This data set includes information on mothers' experiences of disclosing, and maternal reports of parent-child communication about HIV post-disclosure. Apart from data published from Amagugu, little is understood about this age group of HIV-exposed children and their understanding about parental HIV in high prevalence regions (Krauss et al., 2013).

The primary aim of this research is to investigate the content of primary school-aged children's death-related questions post maternal HIV disclosure. Secondary aims investigate maternal and child characteristics associated with children asking death-related questions and potential short term consequences of death-related discussions on children's mental health.

## 2 Methods

### 2.1 Setting

The research was conducted at the Africa Health Research Institute (AHRI) previously known as the Africa Centre for Population Health, or Africa Centre) in South Africa (2010–2012). The area is mostly rural, has a high HIV prevalence and incidence (Zaidi et al., 2013) and a successful HIV Treatment and Prevention programme, with provision of free drugs and clinical care since 2004 (Houlihan et al., 2011).

### 2.2 Design

Amagugu was found to be culturally acceptable and feasible when initially tested in a small pilot study (Rochat et al., 2013). A non-experimental evaluation design was chosen to explore whether this intervention approach was feasible, acceptable and increased rates of disclosure, and to examine factors associated with disclosure in the local context, prior to designing a randomised controlled trial. The design followed the guidelines for complex interventions (Craig et al., 2008) using validated measures, including multiple data collection points, and collecting qualitative data to inform the development of a randomised controlled trial protocol (NCT01922882).

### 2.3 Sample

**Fig. 1:**
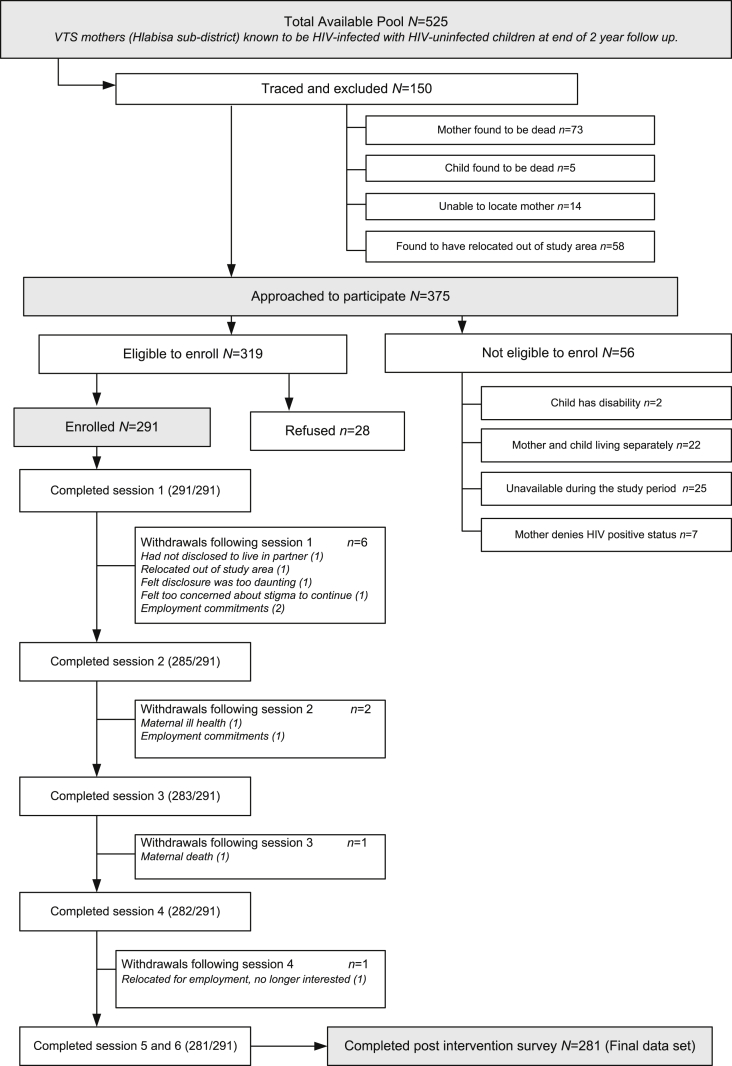
Consort diagram representing participants enrolled into the study.

Amagugu re-enrolled HIV-infected mothers with HIV-uninfected children aged 6–10 years who had been part of a large infant feeding study (Vertical Transmission Study -VTS) previously conducted at the Africa Centre, 2001–2006 (Bland et al., 2010). We approached mothers who at the end of VTS were known to be HIV-infected with an HIV-uninfected child (Fig. 1). VTS mothers had tested for HIV during pregnancy, received antenatal and postnatal counselling, were assessed at two years postnatally, including re-testing for HIV and consenting to be re-contacted for future studies. In this Amagugu study the VTS child was purposely selected as the participating child with whom the mother would undertake the disclosure intervention, given their appropriate age, and known HIV status (HIV-uninfected and aged 6–10 years at enrolment). Additional inclusion criteria required that mother and child were in reasonable physical and mental health; mother was living in the study area with her child, and, if migrant, resided with the child for ≥2 nights per week, to ensure support during the disclosure period.

Of an available pool of 525 mothers 136 (26%) were ineligible (due to death, relocation) and 14 (3%) were un-traceable. Of 375 women approached to participate, 291 (78%) enrolled (see Fig. 1). Amongst enrolees, 10 (3%) withdrew during the study, 281 completed follow-up. The 53 Amagugu non-participators (refusals and not available for participation) were more likely to be younger than participators (*M* 34.7 years; *M* 37.0 years, *p* = 0.003), although the difference was small. No other significant differences were found in maternal IQ, education, employment, relationship status, antiretroviral therapy (ART), child age or gender.

Written informed consent was obtained from mothers and assent from children. Ethical approval was obtained from the Biomedical Ethics Committee of the University of KwaZulu-Natal (Ref: BF 144/010).

### 2.4 Intervention

The content and intervention approach (Rochat et al., 2013), and the conceptual framework (Rochat et al., 2016), have been described elsewhere and are summarised in Supplementary Fig. S1. Avoidant coping is common in HIV-infected mothers and can impact on parenting behaviours (Allen et al., 2014). The intervention aimed to shift the mother to a more active coping style, changing parenting behaviour towards disclosure, health education, and care and custody planning for the child. While structured, the intervention allowed mothers to adjust content (such as level of disclosure) to suit their personal circumstances, readiness and family needs. Lay-counsellors provided training and support to the mother. They did not intervene with children directly, instead the mother communicated with her child independently, promoting parental empowerment.

### 2.5 Data collection

Data were collected at four time points: a baseline assessment including psychometric assessments; two short structured interviews (the first one week after the disclosure event – the “post-disclosure interview”; the second one week following a health promotion visit to the local clinic – the “post-clinic interview”); and an endpoint post-intervention assessment 2–3 weeks after the intervention was completed, where psychometric assessments were repeated. This manuscript reports on data from pre/post assessments and on data collected at the post-disclosure interview. Data collected in the post-clinic interview are published elsewhere (Mkwanazi et al., 2013). Mothers were provided with an airtime voucher to contact the counsellor once disclosure was complete; all 281 mothers completed the post-disclosure interview.

### 2.6 Outcomes

The primary outcome of Amagugu was parental HIV disclosure (full; partial, none) and secondary outcomes included maternal and child mental health and parenting stress, the results of which have been reported elsewhere (Rochat et al., 2014, Rochat et al., 2015).

### 2.7 Measures

#### 2.7.1 Socio-demographic survey

Baseline assessment included a study specific questionnaire collecting socio-demographic data.

#### 2.7.2 Children's questions post-disclosure (post-disclosure interview)

Given the sensitivity of the topic, children's age and vulnerability, and limitations on the reliability of their reporting, we did not conduct interviews with children about their mothers' HIV. Instead, maternal reports of children's reactions to, and questions about, HIV disclosure were collected at the post-disclosure interview. Data were collected during a face-to-face structured interview, conducted by the same IsiZulu-speaking counsellors who implemented the intervention, each with 3–5 years' research experience, and who had received training on data collection. Mothers contacted the interviewer once disclosure had occurred and interviews were scheduled. The interview guide examined maternal reports of the child's initial reactions to disclosure, using fixed categories (calm, confused, surprised, emotional, frightened) drawn from the literature (Murphy et al., 2006). Thereafter, mothers were asked to recall the exact questions children asked post-disclosure, which were recorded verbatim in English using pencil and paper. Given available resources, interviews were not tape-recorded. As part of quality assurance a random selection of interviews were observed by a supervising researcher (IsiZulu speaking PhD student with over 10 years' research experience) who validated the quality of interview note taking against her own interview notes. While data on children's questions relied on maternal recall, the timeframe from disclosure event to interview was relatively short, ranging from 5 to 12 days. The time in days from baseline assessment to the post-disclosure interview was similar across mothers (*M* 62.7 *SE* 3.1 *CI* 56, 68).

#### 2.7.3 Child cognition

Since cognitive capacity might determine a child's capacity to understand disclosure, the Ravens Coloured Progressive Matrices (Ravens-CPM) was completed at baseline. Scores were used as continuous variables in the analysis.

#### 2.7.4 Children's mental health

The parent-report version of the Child Behaviour Checklist (CBCL) (Achenbach and Rescorla, 2001) was completed by mothers pre- and post-intervention. The CBCL is widely validated in many cultural settings, including South Africa (Rescorla et al., 2007), and was translated for this research with a translation licence from the developers. The CBCL scores (including missing values) were transformed and normed using the test developers^®^ASEBA standardised Rating-To-Score (RTS) software. RTS produces normed t-scores for a Total problems score and Internalising and Externalising problem sub-scales. The CBCL had good pre and post-test reliability (Cronbach Alpha Pre *α* = 0.94; Post *α* = 0.92) in this sample. Importantly, mental health problems were not validated using clinical interview methods, these relied only on parent-report, and should be interpreted as an indication of parental perceptions of child risk, rather than an objective measure of child mental health problems.

### 2.8 Data analysis

#### 2.8.1 Qualitative data analysis

**Fig. 2:**
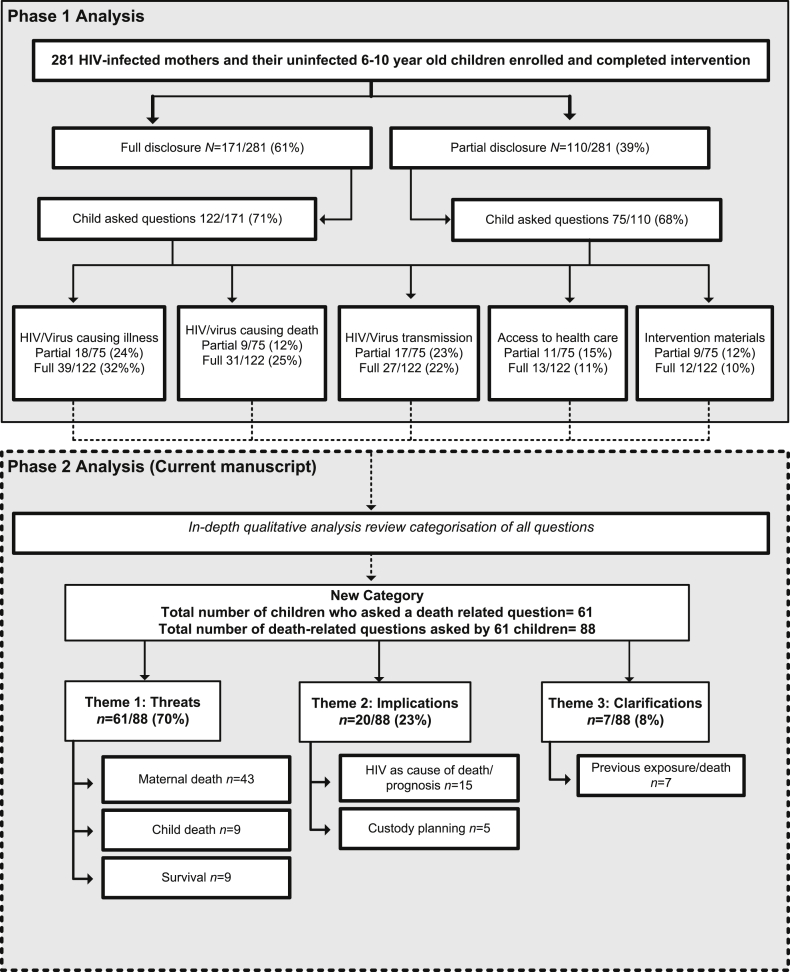
Two phases of qualitative analysis in Amagugu.

Fig. 2 illustrates the two phases of qualitative analysis. Previously, in Phase 1 we analysed and reported on children's post-disclosure questions using content analysis (Rochat et al., 2014). The qualitative analysis was undertaken using NVivo (QSR International Pty Ltd. Version 10, 2012). A total of 197/281 children asked questions, the purpose of the Phase 1 analysis was to categorize the types of questions asked, and to quantify the number of responses under each category. Repeated readings of the data on child questions (by the second and third author) led to the coding of recurrent words and phrases as they appeared in the written post-disclosure interview forms. Codes were reviewed and discrepancies resolved together with the first and last author, and allocated to categories, based on how they informed or differed from each other. The latter analysis identified 40 children who asked a question directly about maternal death, using the words ‘death, dead or die’ (Rochat et al., 2014).

In Phase 2 we re-examined all children's questions using NVivo, to broaden the scope and interpretive possibilities of our previous analysis, while remaining close to the manifest content of the dataset. Data were analysed by the third author using content analysis; an inductive approach to category generation was used (Gläser and Laudel, 2013). The steps included a review of the literature on children with parents with terminal illness to draw up a list of key words; this list was broader than the original code list in Phase 1 and included “death”, “dying”, “died”, “kill/ed”, “end of life”, “survive”, “live”, and “alive”. Other culturally relevant death-related words such as “passing” or “being late”, locally representing “having died” were included. An important distinction in the Phase 2 code list was the inclusion of codes to identify instances where children asked questions about illness and death as survival questions, for example ‘will you still be alive after having HIV’. The code list was reviewed together with the first and third authors, finalised, and used as criterion by which material was included or excluded from the revised death-related question category. Following Phase 2 analysis, 61 children were identified to have asked a death question. Thereafter questions were subjected to a thematic analysis (Huberman and Miles, 2002), by the third author resulting in three themes. Themes were independently verified and confirmed by the first and second author. Z test for proportions were used to examine gender differences in themes (two tailed, significance *p* = 0.050).

#### 2.8.2 Quantitative analysis

Data were analysed using STATA13. Children were grouped by whether they asked a death-related question or not. Using the “asked death-related question” variable as a binary outcome, we used logistic regression to examine the maternal and child characteristics associated with this variable. In the maternal model the independent variables were based on previous work (Rochat et al., 2014), including: maternal age, education, employment, access to regular remittance; CD4, HIV treatment status and hospitalisations; and level of disclosure level (full vs. partial). For children, the model included the child's age, gender, prior hospitalisation, whether the mother had reported that the child had a fearful reaction to disclosure, and Raven-CPM score (all known to be associated with understanding of death in the literature or in Phase 1 results) and level of disclosure (full vs. partial). We tested a combined model controlling first for significant variables only, and thereafter for all variables. We tested for differences in post-intervention CBCL outcomes by “asked death-related question” group using linear regression, controlling for pre-intervention CBCL scores and for variables significant in maternal and child models. We also tested this for children who scored above the clinical threshold of ≥65 on the CBCL.

## 3 Results

A total of 197/281 children (Fig. 1) were reported to have asked a question post-disclosure; of whom 61/197 (31%) asked a death-related question. Amongst these 61 children, some asked more than one question (88 questions in total). The ratio of gender to number of questions showed that girls (31:49) asked more questions than boys (30:39), but these differences were not significant (Table 1).

### 3.1 Qualitative analysis: categories of death-related questions asked by children

Qualitative analysis resulted in six question categories. These categories were grouped into three themes: ‘*threats’* (of maternal death/child death/survival); *‘implications’* (HIV causes death/prognosis, custody planning); *‘clarifications’* (previous exposure/deaths) (Fig. 2).

Table 1 outlines the three themes, with their categories, disaggregated by gender and including examples of child questions. Most children (of both genders) asked the mother directly if she would die as a consequence of HIV. Girls tended to ask more questions than boys about their mothers' survival and to clarify previous exposure to death, while both boys and girls focused on the mechanisms by which HIV might cause illness and death, and steps to ensure survival. Overall the differences in proportion of questions amongst boys and girls were not significant. Girls’ questions appeared to reflect more relational concerns linked to people, while boys were somewhat more pragmatic.

Most children asked questions demonstrating pre-operational and some logical thinking, with questions becoming more complex and abstract in nature with increasing age.

*“Why are you telling me that the virus is a killer, but that it will not kill you?” (ID-256, full disclosure, child 6 years)*

Younger children's questions frequently focused on attempting to clarify previous experiences of death with new information provided by the mother, for example:

*“Does my stepfather have it? Did my father have the virus because I saw that he was also taking pills before he died?” (ID-21, partial disclosure, child 6 years)*

Amongst younger children logical errors were common, illustrating developmentally appropriate difficulties with abstract information related to viruses and illness, for example:

*“Do I have HIV because sometimes I get a cough?” (ID-222, full disclosure, child 6 years)*

Younger children also had difficulties with the concept of reversibility and death being universal, and often sought clarification around cause and effect, for example:

*“Why are you not dead or sick like other people? Why are you just pretty as you are, even though you have HIV?” (ID-31, full disclosure, child 7 years old)*

With increasing age children began to ask questions regarding the social and familial context of HIV and requested clarification about disclosure of illness to others, illustrating the mastery of a more mature understanding of death.

*“Does my father have HIV? What caused my grandfather to die? Do my uncles have HIV? Why don't you call everybody in the house and tell them too? (ID-103, full disclosure, child 7 years)*

Older children aged 8–9 years appeared to have a better grasp of the notion that death was universal, irreversible and final thus signalling changes in child care context, leading children to seek guidance and reassurance, for example:

*“Who will take care of us when you are dead?” (ID-130, full disclosure, child 8 years)*

Similarly, older children introduced questions which explored the relational context of HIV infection and the connection between treatment and survival, for example:

*Why you fell in love with my father? Did you get it (HIV) from my father or other men? Would my father be still alive if he had taken medicine? (ID-16, partial disclosure, child 8 years)*

Only a few older children demonstrated the capacity to engage with a mature concept of death:

*“What will it be like for you when you die? How long are you going to live?”(ID-213, full disclosure, child 9 years)*

### 3.2 Quantitative analysis: characteristics associated with, and consequences of asking a death-related question

In Table 2 we show that mothers without a source of regular remittance were 75% less likely to report that their child asked a death-related question in multivariate analysis (*AOR* 0.25 [*CI* 0.10, 0.59] *p* = 0.002).

Maternal full disclosure was significantly associated with a death question being reported, but only in univariate analysis (*OR* 2.60 [*CI* 1.35, 5.0] *p* = 0.004).

In the second model (see Table 3) we examined child characteristics associated with reporting death-related questions, finding that children's age, gender, and cognition scores were not significantly associated, while full versus partial disclosure significantly increased odds of the child asking a death-related question (*AOR* 2.55 [*CI* 1.28, 5.06] *p* = 0.008), as did maternal reports that the child's reaction to disclosure was ‘frightened’ (*OR* 6.16 [*CI* 2.73, 13.92] *p*=<0.001; *AOR* 6.57 [*CI* 2.75, 15.70] *p*=<0.001) although numbers were small and the confidence interval wide.

When only significant variables from the two models (level of disclosure, frightened reaction and regular remittance) were tested in a multivariate model, all three remained significant (Full disclosure *AOR* 2.53 [*CI* 1.27, 5.04] *p* = 0.008; Frightened reaction *AOR* 6.49 [*CI* 2.76, 15.28 *p*=<0.001; No regular remittance *AOR* 0.39 [*CI* 0.21, 0.75] *p* = 0.004). However when controlling for all maternal and child characteristics (see Supplementary Table S1) only the association between death-related questions and access to regular remittance (No remittance *AOR* 0.15 [*CI* 0.05, 0.42] *p*=<0.001) and fearful response (Frightened reaction *AOR* 15.47 [*CI* 3.75, 63.86] *p*=<0.001) remained significant.

### 3.3 Death-related communication and children's mental health

All children showed reductions in CBCL scores post-intervention. Examining group differences post-intervention, by asked a death question, and controlling for baseline, we found no significant differences: Internalising problems (No death question *M* = 50.5; Death question *M* = 51.5 β -0.95 (*t* -0.78) *p* = 0.438); Externalising (No death question *M* = 50.7; Death question *M* = 49.8 β -0.89 (*t* -0.66) *p* = 0.509); Total problems (No death question *M* = 48.9; Death question *M* = 48.8 β -0.96 (*t* -0.07) *p* = 0.944). When we tested this model controlling for regular remittance, fearful response and level of disclosure, and using the clinical threshold ≥65 we again found no significant associations with death question group and total, internalising or externalising problems at the post intervention assessment.

## 4 Discussion

Our findings are in line with literature which has shown that children can develop a good understanding of the causal relationship between a biological disease process (such as HIV) and the threat of death, by middle childhood (Slaughter, 2005). Furthermore, children as young as five years begin to develop capacity to understand disease concepts, and how disease affects bodily organs and function, including potential threats to survival (Hunter and Smith, 2008).

Other HIV disclosure research (Kirshenbaum and Nevid, 2002) found that while mothers reported disclosing when their children were 7–8 years, they acknowledged that their children had been aware of illness-related information for at least 2–3 years prior to the disclosure. For many children and families HIV disclosure emerges as a staged process (Lesch et al., 2007).

The content of children's questions, and the finding that approximately a third of children asked about death specifically, suggests some understanding of the connection between HIV and death, and to some extent, an understanding of concepts of applicability and causation (Slaughter and Griffiths, 2007). This concurs with evidence suggesting that while children may not be able to fully grasp a mature concept of death before the ages of 9–11 years (Labrell and Stefaniak, 2011), their capacity to develop a biological life theory emerges much earlier (Christ and Christ, 2006, Slaughter and Griffiths, 2007). There is increasing recognition that children's exposure to (Hunter and Smith, 2008), and education about (Vlok and de Witt, 2012), illness and death informs their capacity to develop sound conceptualisations of inevitability, applicability, irreversibility, cessation and causation concepts. Children exposed to familial illness and deaths appear to more rapidly assimilate these concepts than children not exposed to familial deaths (Bonoti et al., 2013).

The content of children's questions suggests that they had a high level of exposure to illness and death prior to this study. That HIV treatment only became widely available in the study community in recent years might explain why several children sought to resolve discord between their previous experiences about HIV infection, as implying certain death, with new information that with appropriate care and treatment, survival was possible. It is important, in contexts where exposure to death is high, that children are provided with timely, age-appropriate educational information (Vlok and de Witt, 2012) since it has been shown that increased understanding of death reduces fears in 4–8 year old children (Slaughter and Griffiths, 2007).

Given the prospect of separation through death, children sought information on whether continued access to the life-sustaining resources of their caregiving environment could be expected, how long their mothers might survive, and an indication of custody plans following death. These questions reflect children's capacity to understand the irreversibility concept of death (Slaughter and Griffiths, 2007). There is substantial literature showing that custody planning amongst HIV-infected parents is protective (Mason, 2007) and our data on children's questions indicated a need for this.

Our results showed some limited evidence of different concerns by gender. Girl children tended to have more concerns, in particular about the threats to survival and clarifying exposure to previous death, particularly death within the family. This is a common finding in the literature (Bener et al., 2016, Jordan, 2005) where girls, compared to boys, tend to ask more questions (specifically in relation to applicability and irreversibility) and have greater fears about the threat of death in childhood (Hunter and Smith, 2008, Slaughter and Griffiths, 2007). Although girls appeared to have more fears, boys’ questions also illustrated concerns, and a need for information and reassurance. These findings add weight to the need for additional interventions to support all primary-school-aged children, regardless of gender; particularly as increased understanding is known to reduce fear and anxiety (Slaughter and Griffiths, 2007).

There is often a mismatch between what caregivers believe children can understand, and what children actually understand (Gaab et al., 2013). Their willingness to communicate about HIV-related illness and death may be influenced by cultural beliefs and practices, concerns about stigma, and a desire to protect children (Mdleleni-Bookholane et al., 2004, Van der Heijden and Swartz, 2010). This lack of communication is unhelpful, and can even be harmful for children (Gaab et al., 2013). Our research finds that the period following disclosure is an opportune time to provide children with accurate, developmentally-appropriate information, which would be protective (Dunning, 2006) and prevent the formation of an incomplete or erroneous understanding of HIV, that may compromise children's longer-term adaptation to parental illness and death (Christ and Christ, 2006).

In our examination of the characteristics associated with death-related questions, the finding that the level of disclosure (in particular full disclosure) significantly increased odds of a death-related question post-disclosure, makes intuitive sense, and is aligned to previously reported work (Rochat et al., 2014, Rochat et al., 2015). We show that the reaction of the child to disclosure being reported as fearful was significantly associated with death questions, more so than a full disclosure in and of itself. This finding likely relates to children's previous exposure to HIV-related deaths and illustrates that the threat of death raises concern and anxiety in children, for which they need support and reassurance. However this result should be interpreted with caution given small numbers in this category.

The finding that children whose mothers did not have access to a regular remittance were significantly less likely to ask a death-related question is more complex, particularly as it is unrelated to maternal education or employment. Regular remittances, in settings such as this community (Bigombe and Khadiagala, 2004), are distinct from employment as a source of income. These usually reflect external sources of income from extended family members living elsewhere for migrant work, also from biological parents living separately from their children, as a result of estrangement or lobola (bridal wealth) cultural practices (Rudwick and Posel, 2014). While we do not have sufficient data to test this hypothesis, it is plausible that regular remittances introduce a level of stability and financial security which in turn may be associated with increased maternal agency and control (Govender and Moodley, 2004). This stability coupled with less preoccupation and potentially improved maternal sensitivity (Evans et al., 2008) may contribute to an environment in which children are better able to engage and ask sensitive questions that they might be unable to do in the context of chronic financial insecurity.

While not significant, in line with other findings, we show similar patterns of increasing age and exposure to death increasing children's understanding of death (Hunter and Smith, 2008). Literature on the role of cognition in children's understanding is somewhat contradictory (Hunter and Smith, 2008), with some studies finding associations and others not. In our research child cognition was not significantly associated with children asking about death; however this may be a result of the small sample size.

We have previously reported that post-intervention both mothers and their children showed improved mental health (Rochat et al., 2015). In this research, we show that when disclosure includes communication about death, this did not have any significant immediate negative consequences, in the short-term, as measured by maternal report, a few weeks post-disclosure at the follow up assessment. This concurs with existing evidence that children's emotional distress following disclosure is generally reported to be short-lived (Krauss et al., 2013). Longer term follow up is required to interrogate this issue thoroughly.

Caregiver's hesitance about talking with children about illness and death is not uncommon in the literature (Gaab et al., 2013). However, this may limit children's ability to form a comprehensive and accurate understanding of HIV infection and the possibility of death, potentially leaving children partially aware, but also uncertain, fearful and without easy recourse for reassurance from adults (Stein et al., 2009). In our intervention, content was designed to support mothers to prepare for these questions about death which may have mitigated the negative effects of the disclosure and discussions about death on children's emotional wellbeing. Mothers were encouraged to provide information to the child about the life-extending capacity of ART, and that the mother's adherence to treatment would reduce the immediate threat of death. Providing this information to the child, prior to parental illness, may increase resilience (Saldinger et al., 2004a, Saldinger et al., 2004b), particularly in light of evidence that the pre-death period may be most challenging for HIV-exposed children Chi and Li, (2013).

### 4.1 Limitations

This research is limited by the lack of a controlled design, relatively small sample size, and that all child data was based on maternal report and recall. The uncontrolled design limits our ability to know how children who were not part of the intervention would react to disclosure. An further limitation of this research is that emotional and behavioural data relied solely on a maternal report rating scale, and while the CBCL is very well validated, these data can only be interpreted to show that maternal perceptions of children's problems reduced, not that children's’ actual emotional problems reduced, because these were not objectively measured. The disclosure process may have changed maternal sensitivity to reporting emotional and behavioural symptoms in children. Furthermore, given that CBCL assessment occurred soon after disclosure, we do not know the longer term effects of disclosure on children's mental health. Importantly while the content of children's questions suggests indirectly that children had an awareness of HIV and the threat of parental death, we are unable to determine whether children had a full understanding of death, as determining this would require more direct interviewing and assessment of children. Limited resources also prevented us from measuring sibling effects. Controlled studies, with longer term follow up, which include clinical interviews with children, are needed.

## 5 Conclusion

We find that maternal disclosure, in particular full disclosure, prompts discussion on death with about a third of children. The extent to which this occurs appears to be contextualised by whether the child has a fearful response to disclosure (perhaps linked to previous exposures) and by the financial and social stability of the family environment which could support children to feel comfortable to talk about sensitive issues. Communication about death did not have immediate negative consequences, at least by maternal report. Our research illustrates that HIV-exposed uninfected children have similar information needs to children affected by parental illness and death outside the context of HIV. Providing for the informational and educational needs of HIV-exposed children is an important public health priority.

## Figures and Tables

**Table 1:** Endorsement of thematic categories by children, stratified by gender, with example of questions asked.

	Boys^*^N (%)	Boys example questions	Girls^*^N (%)	Girls example questions	*Z (p)*
**Theme 1: *“Threats”***					
Maternal death	21/30 (70%)	Are you going to die? Are you not going to die then? Can I cry because you are going to die soon? When you were sick before were you going die?	22/31 (71%)	Are you going to die, Mom? If you get sick then will you die? Does HIV kill you? When are you going to die? Are you still going to be alive? Why are you not sick or dead?	*Z* = -0.082 *p* = 0.936
Child death	5/30 (17%)	If I happen to get the virus will I die or am I going to be alive like you? What about us, are we going to die? How can you be sure that I am HIV negative? If I get sick am I going to die? Do I have HIV too?	4/31 (13%)	How did my brother and I survive? Are we (children) going to have HIV? Do I also have HIV? What am I supposed to do when I am sick, too not die?	*Z* = 0.414 *p* = 0.681
Survival	2/30 (7%)	How do you survive because all people suffering from HIV are dying? What will happen if you don't find nurses and tablets at the clinic?	7/31 (23%)	How long are you going to living still? Are you going to survive? Are you going to survive by taking tablets? Are you sure that tablets are helping you? How long are going to survive? Is it curable? How long are you going to live? Are you going to die if you give birth to a new born?	*Z* = -1.752 *p* = 0.0801
**Theme 2:** “***Implications”***					
HIV as cause of death/prognosis	7/30 (23%)	Are the pills going to make you live long, Mum? If you didn't get the pills would you be dead? Do all viruses kill? If someone does not take pills when will her life end? If you don't take the pills are you going to die? Does HIV get cured like all other viruses like TB? How do I behave to make sure I don't get HIV?	8/31 (26%)	If you get sick then will you die? Does a person die if they have HIV? If a person has a virus, do they die? When does a person die if they have HIV? Is HIV incurable? Who died of HIV, why couldn't they go to the clinic? As you are eating pills that means you will not die? What if the pills are not working or fail to work?	*Z* = -0.224 *p* = 0.825
Custody planning	3/30 (10%)	What about us and my father when you die? If you die of HIV who is going to care for us? Does it mean that I will not have both parents?	2/31 (7%)	If you die Mom who will care for us? What we may be like when you die?	*Z* = 0.505 *p* = 0.610
**Theme 3: *“Clarifications”***					
Previous exposure/death	1/30 (3%)	Did my father have AIDS because he was taking pills before he died?	6/31 (19%)	What caused my dad to die? What caused my sister to die? What caused my grandfather to die? What about my father's other children because they are late? Would my father be still alive if he took treatment? What caused my brother to die?	*Z* = -1.96 *p* = 0.050

^∗^Note: some children asked more than one question, hence total responses for girls was 49 questions by 31 girls; 39 questions by 30 boys.

**Table 2:** Maternal characteristics associated with maternal report of children having asked death-related questions.

Characteristics	No death question *N* = 220	Death question *N* = 61	Univariate *OR (95%CI) p*	Multivariate *AOR (95%CI) p*
**Maternal age**				
Median (Inter-quartile Range)	35 (24–54)	34 (23–48)	0.99 (0.96, 1.04) *p* = 0.986	1.05 (0.98, 1.12) *p* = 0.175
**Maternal education (%)**				
No education	83 (37.73)	25 (40.90)	1 (1.0, 1.0)	1 (1.0, 1.0)
Completed some or all primary	114 (51.82)	34 (55.74)	0.99 (0.55, 1.78) *p* = 0.974	0.79 (0.34, 1.83) *p* = 0.586
Completed some or all secondary	4 (1.82)	7 (1.64)	0.83 (0.89, 7.77) *p* = 0.870	1.70 (0.12, 24.81) *p* = 0.697
Post school education	16 (7.27)	7 (1.64)	0.21 (0.03, 1.64) *p* = 0.136	0.15 (0.01, 1.68) *p* = 0.124
Missing	3 (1.36)	0 (0)	–	–
**Maternal employment (%)**				
Employed	72 (32.73)	18 (29.51)	1 (1.0, 1.0)	1 (1.0, 1.0)
Unemployed	145 (65.91)	43 (70.49)	1.18 (0.64, 2.20) *p* = 0.588	1.16 (0.50, 2.78) *p* = 0.735
Missing	3 (1.36)	0 (0)	–	–
**Maternal regular remittance (%)**				
Receives regular remittance	48 (21.82)	25 (40.98)	1 (1.0, 1.0)	1 (1.0, 1.0)
Does not receive regular remittance	172 (78.18)	36 (59.02)	0.52 (0.32, 0.84) *p* = 0.003**	0.25 (0.10, 0.59) *p* = 0.002**
**Maternal CD4 count (most recent) (%)**				
≤350	60 (27.3)	17 (27.87)	1 (1.0, 1.0)	1 (1.0, 1.0)
351–500	37 (16.8)	16 (26.23)	1.53 (0.69, 3.38) *p* = 0.298	1.61 (0.63, 4.07) *p* = 0.317
≥501	59 (26.8)	12 (19.67)	0.72 (0.32, 1.63) *p* = 0.429	0.60 (0.24, 1.49) *p* = 0.267
Missing	64 (29.1)	16 (26.23	–	–
**Maternal hospitalisation (past year) (%)**				
Yes	23 (10.45)	7 (11.48)	1 (1.0,1.0)	1 (1.0, 1.0)
No	196 (89.09)	54 (88.52)	0.91 (0.37, 2.22) *p* = 0.828	1.62 (0.46, 5.68) *p* = 0.452
Missing	1 (0.45)	0 (0)	–	–
**Maternal HIV treatment status (%)**				
On ART	93 (42.27)	25 (40.98)	1 (1.0, 1.0)	1 (1.0, 1.0)
Not on ART	122 (55.45)	33 (54.10)	1.01 (0.56, 1.81) *p* = 0.983	0.88 (0.39, 2.0) *p* = 0.766
Missing	5 (2.27)	3 (4.92)	–	–
**Maternal perception of current health (%)**				
My health is excellent				
False	64 (29.22)	19 (31.15)	1 (1.0, 1.0)	1 (1.0, 1.0)
True	155 (70.78)	42 (68.85)	0.91 (0.49, 1.69) *p* = 0.771	0.77 (0.35, 1.70) *p* = 0.516
**Mothers disclosure level (%)**				
Partial	96 (43.64)	14 (22.95)	1 (1.0, 1.0)	1 (1.0, 1.0)
Full	124 (56.36)	47 (77.05)	2.60 (1.35, 5.0) *p* = 0.004**	2.05 (0.91, 4.62) *p* = 0.083

**Table 3:** Child characteristics associated with maternal report of children having asked death-related questions.

Characteristics	No death question *N* = 220	Death question *N* = 61	Univariate *OR (95%CI) p*	Multivariate *AOR (95%CI) p*
**Child gender (%)**				
Female	109 (49.55)	31 (50.82)	1 (1.0, 1.0)	1 (1.0, 1.0)
Male	111 (50.45)	30 (49.18)	0.95 (0.54, 1.68) *p* = 0.860	0.85 (0.46, 1.57) *p* = 0.604
**Child age**				
Median (Inter-quartile range)	7 (5–10)	7 (6–9)	1.11 (0.84, 1.47) *p* = 0.461	1.04 (0.75, 1.44) *p* = 0.833
**Child ravens scores**				
Mean (*SD*)	15.74 (5.1)	15.91 (6.4)	1.01 (0.95, 1.06) *p* = 0.832	0.99 (0.93, 1.05) *p* = 0.803
**Mothers disclosure level (%)**				
Partial	96 (43.64)	14 (22.95)	1 (1.0, 1.0)	1 (1.0,1.0)
Full	124 (56.36)	47 (77.05)	2.60 (1.35, 5.0) *p* = 0.004**	2.55 (1.28, 5.06) *p* = 0.008**
**Child hospitalisation (past year) (%)**				
Yes	33 (15.00)	10 (16.39)	1 (1.0, 1.0)	1 (1.0,1.0)
No	175 (79.55)	46 (75.41)	0.87 (0.40, 1.89) *p* = 0.720	1.01 (0.43, 2.41) *p* = 0.976
Missing	12 (5.45	5 (8.20)		
**Child frightened reaction post-disclosure (%)**				
No	208 (82.21)	45 (17.79)	1 (1.0, 1.0)	1 (1.0,1.0)
Yes	12 (42.86)	16 (57.14)	6.16 (2.73, 13.92) *p*=<0.001***	6.57 (2.75, 15.70) *p*=<0.001***
