# Supplementary material for: Communication about HIV and death: Maternal reports of primary school-aged children's questions after maternal HIV disclosure in rural South Africa
Source: Soc Sci Med. 2017 Jan;172:124–34. doi: 10.1016/j.socscimed.2016.10.031 (PMC5224234; doi:10.1016/j.socscimed.2016.10.031)
Supplement: Supplementary file 1 [file mmc1.docx]

Figure S1. Overview of intervention model and approach

*Note*: Figure reproduced with permission from: Rochat, T., Mitchell, J., Stein, A., Mkwanazi, N.B., & Bland, R.M. (2016). The Amagugu Intervention: A conceptual framework for increasing HIV disclosure and parent-led communication about health among HIV-infected parents with HIV-uninfected primary school-aged children. Front Public Health, 4, 183
