# Supplementary material for: Communication about HIV and death: Maternal reports of primary school-aged children's questions after maternal HIV disclosure in rural South Africa
Source: Soc Sci Med. 2017 Jan;172:124–34. doi: 10.1016/j.socscimed.2016.10.031 (PMC5224234; doi:10.1016/j.socscimed.2016.10.031)
Supplement: Supplementary file 2 [file mmc2.docx]

Table S1. Combined Model containing Maternal and child characteristics associated with maternal report of children having asked death-related questions

| **Characteristics** | **No death question *N*=220** | **Death question**  ***N*=61** | **Univariate**  ***OR (95%CI) p*** | **Multivariate**  ***AOR (95%CI) p*** |
| --- | --- | --- | --- | --- |
| ***Maternal Age*** |  |  |  |  |
| Median (Inter-quartile Range) | 35 (24-54) | 34 (23-48) | 0.99 (0.96, 1.04) *p*=0.986 | 1.02 (0.95, 1.10) *p*= 0.583 |
| ***Maternal Education (%)*** |  |  |  |  |
| No education | 83 (37.73) | 25 (40.90) | 1 (1.0, 1.0) | 1 (1.0, 1.0) |
| Completed some or all primary | 114 (51.82) | 34 (55.74) | 0.99 (0.55, 1.78) *p*=0.974 | 0.63 (0.23, 1.70) *p*=0.359 |
| Completed some or all secondary | 4 (1.82) | 7 (1.64) | 0.83 (0.89, 7.77) *p*=0.870 | 2.30 (0.12, 44.78) *p*=0.582 |
| Post school education | 16 (7.27) | 7 (1.64) | 0.21 (0.03, 1.64) *p*=0.136 | 0.22 (0.02, 3.11) *p*=0.261 |
| Missing | 3 (1.36) | 0 (0) | - | - |
| ***Maternal Employment (%)*** |  |  |  |  |
| Employed | 72 (32.73) | 18 (29.51) | 1 (1.0, 1.0) | 1 (1.0, 1.0) |
| Unemployed | 145 (65.91) | 43 (70.49) | 1.18 (0.64, 2.20) *p*=0.588 | 1.63 (0.57, 4.66) *p*=0.359 |
| Missing | 3 (1.36) | 0 (0) | - | - |
| ***Maternal Regular Remittance (%)*** |  |  |  |  |
| Receives regular remittance | 48 (21.82) | 25 (40.98) | 1 (1.0, 1.0) | 1 (1.0, 1.0) |
| Does not receive regular remittance | 172 (78.18) | 36 (59.02) | 0.52 (0.32, 0.84) *p*=0.003** | 0.15 (0.05, 0.42) *p*=<0.001*** |
| ***Maternal CD4 Count (most recent) (%)*** |  |  |  |  |
| ≤350 | 60 (27.3) | 17 (27.87) | 1 (1.0, 1.0) | 1 (1.0, 1.0) |
| 351-500 | 37 (16.8) | 16 (26.23) | 1.53 (0.69, 3.38) *p*=0.298 | 1.32 (0.43, 4.01) *p*=0.630 |
| ≥501 | 59 (26.8) | 12 (19.67) | 0.72 (0.32, 1.63) *p*=0.429 | 0.74 (0.25, 2.18) *p*=0.583 |
| Missing | 64 (29.1) | 16 (26.23 | - | - |
| ***Maternal Hospitalisation (past year)* *(%)*** |  |  |  |  |
| Yes | 23 (10.45) | 7 (11.48) | 1 (1.0,1.0) | 1 (1.0, 1.0) |
| No | 196 (89.09) | 54 (88.52) | 0.91 (0.37, 2.22) *p*=0.828 | 1.43 (0.34, 6.09) *p*=0.628 |
| Missing | 1 (0.45) | 0 (0) | - | - |
| ***Maternal HIV Treatment Status (%)*** |  |  |  |  |
| On ART | 93 (42.27) | 25 (40.98) | 1 (1.0, 1.0) | 1 (1.0, 1.0) |
| Not on ART | 122 (55.45) | 33 (54.10) | 1.01 (0.56, 1.81) *p*=0.983 | 0.74 (0.27, 2.0) *p*=0.549 |
| Missing | 5 (2.27) | 3 (4.92) | - | - |
| ***Maternal Perception of Current Health* (*%*)** |  |  |  |  |
| My health is excellent |  |  |  |  |
| False | 64 (29.22) | 19 (31.15) | 1 (1.0, 1.0) | 1 (1.0, 1.0) |
| True | 155 (70.78) | 42 (68.85) | 0.91 (0.49, 1.69) *p*=0.771 | 0.54 (0.21, 1.36) *p*=0.189 |
| ***Mothers Disclosure Level* (*%*)** |  |  |  |  |
| Partial | 96 (43.64) | 14 (22.95) | 1 (1.0, 1.0) | 1 (1.0, 1.0) |
| Full | 124 (56.36) | 47 (77.05) | 2.60 (1.35, 5.0) *p*=0.004** | 2.36 (0.91, 6.12) *p*=0.077 |
| ***Child Gender* (*%*)** |  |  |  |  |
| Female | 109 (49.55) | 31 (50.82) | 1 (1.0, 1.0) | 1 (1.0, 1.0) |
| Male | 111 (50.45) | 30 (49.18) | 0.95 (0.54, 1.68) *p*=0.860 | 0.63 (0.25, 1.57) *p*=0.321 |
| ***Child Age*** |  |  |  |  |
| Median (Inter-quartile range) | 7 (5-10) | 7 (6-9) | 1.11 (0.84, 1.47) *p*=0.461 | 1.20 (0.76, 1.91) *p*=0.435 |
| ***Child Ravens Scores*** |  |  |  |  |
| Mean (*SD*) | 15.74 (5.1) | 15.91 (6.4) | 1.01 (0.95, 1.06) *p*=0.832 | 0.98 (0.90, 1.05) *p*=0.541 |
| ***Child Hospitalisation (past year) (%)*** |  |  |  |  |
| Yes | 33 (15.87) | 10 (17.86) | 1 (1.0, 1.0) | 1 (1.0,1.0) |
| No | 175 (84.13) | 46 (82.14) | 0.87 (0.40, 1.89) *p*=0.720 | 1.06 (0.34, 3.27) *p*=0.926 |
| ***Child Frightened Reaction Post-Disclosure (%)*** |  |  |  |  |
| No | 208 (82.21) | 45 (17.79) | 1 (1.0, 1.0) | 1 (1.0,1.0) |
| Yes | 12 (42.86) | 16 (57.14) | 6.16 (2.73, 13.92) *p*=<0.001*** | 15.47 (3.75, 63.86) *p*=<0.001*** |
