# Supplementary material for: Communication about HIV and death: Maternal reports of primary school-aged children's questions after maternal HIV disclosure in rural South Africa
Source: Soc Sci Med. 2017 Jan;172:124–34. doi: 10.1016/j.socscimed.2016.10.031 (PMC5224234; doi:10.1016/j.socscimed.2016.10.031)
Supplement: Supplementary file 3 [file mmc3.pdf]

because the child was coping well and they felt more confident. If the mother chooses the word 'virus', the rhyme can be learnt using *Virus, Virus, Virus....*

The important thing about using the word 'virus' instead of 'HIV' is that the mother should still tell the child she is infected with a virus at the end of the Body Map exercise, otherwise she has not disclosed. It is also important not to mislead the child to think that the mother has another virus or an illness like TB. By misleading the child you are breaking your trust with them. At some point they will learn that the parent does in fact have 'HIV' and it will hurt them very much that their parents did not tell them the truth.

IMPORTANT LEARNING TIP:

It is not correct for the mother to tell the child that she has a different virus or illness to 'HIV', for example to tell the child that she has TB or a cold. Children aged 6-10 years find it very difficult later on if they find out that their parent did not tell them the truth about something. The child needs to know, and they need to know while their mother is healthy, so that she can support them and help them to learn.

At this point in the training you will need to be both firm and confident with mothers. You have to show them that you believe in them and help them feel this is the best thing to do. Remember your Talking Points for Session 1 and, if you need to, remind the mother of them!

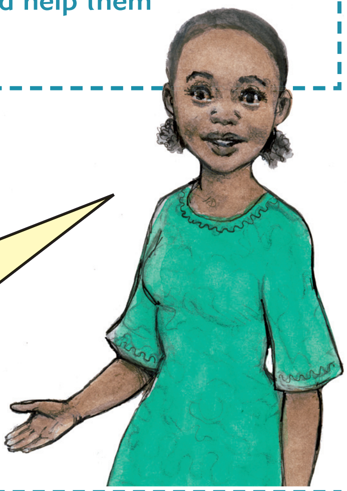

IMPORTANT LEARNING TIP:

Initially some mothers have difficulty remembering all the steps. It is partly because they need time to learn them, but they may also feel a bit overwhelmed and filled with dread about actually disclosing. It's important to push forward and help the mother to practise, because by 'doing' and practising, we move through the anxiety. Some mothers may need a few practice sessions before they feel confident.

Preparing the mother for the child's reactions

Almost all the children in the demonstration project responded calmly to disclosure and only a few were emotional and tearful. Remind mothers of this and explain that what they imagine might happen is usually far worse than what actually happens. For many mothers the opposite reaction was what shocked them the most – they expected the child to be upset but, instead, the child was fine and happy to be talking and learning about HIV.

There are some common questions which children usually ask, and in the next section we will offer some suggested answers for them. Some children ask one or two questions and then move on to the Playing Cards. Later they may ask more questions. Sometimes it will be a day or two before they ask for more information – that is all normal.

Common questions children ask after disclosure:

HIV information questions

In the demonstration project we found that children usually had further questions about how HIV works in the body – and that they tended to ask these first before talking about the mother being HIV-positive. It is reasonable that mothers can expect that. These questions often include things like:

- *Does HIV live in your body forever?*
- *How long does HIV take before it makes you sick?*

The important rule is to make sure that the answers are close to the truth.Encourage the mother to avoid dwelling on things and making them into bigger issues than they are. Children are very straight-forward – they ask you what they want to know about. You should answer the question and move on. If you think the child is asking something because they feel worried about it, it's important to offer reassurance.

So, for example, if a child asks:

- *Does HIV live in your body forever?*

The mother could answer something like:

Yes, it does. There is nothing that makes HIV go away completely. In that way it's not like a cold – it stays. But that is okay because it can be kept under control by medicine and by taking care of yourself. You know that I take good care of myself. I also want to take care of you and that's why I have told you what is going on so that you don't worry if you see me going to clinic or taking my tablets. As you can see, I am fine and together as a family we are going to make sure I stay fine – okay!

Other talking tips for 6-10 year old children about HIV include:

- *How long before you get sick?*

It is different for each person, but part of staying healthy is not to worry too much about being sick when you are not sick. As you can see I am not sick, and later today I am going to teach you about the things I do to stay healthy – things that you can help me with.

- *Can HIV be cured?*

No, at the moment there is no cure, but the doctors have found ways to treat HIV, just like they treat other illnesses like asthma or diabetes. HIV does not go away but we can treat it so that we can live a long life.

This a good place to move on to the Playing Cards if the child has no other questions, so the mother could say something like:
I have a card game which teaches you more about how to stay healthy. Can I show you? It might make you worry less about how I keep myself healthy.

HIV status questions

It is very common for children to ask whether they are infected with HIV, or whether other family members have HIV. Usually a child will ask if they are infected first, and the good news is that the mother can offer good news and say' no' and reassure the child. This is also a chance for the mother to say something positive to the child like:

When you were born I was very careful to make sure you were not infected with HIV because I love you very much and I didn't want you to have HIV like I did.

Children may also sometimes ask about other people in the family. This is especially the case if there are other adults or children who have been sick or on treatment in recent months. Children may ask things like:

- *Does my sister/uncle/father also have HIV? Is that why s/he takes tablets every day?*

As long as the mother is not violating the wishes of any other adults in the household it is better if she responds with honesty and confirms that other family members are also infected. She could say something like:

Yes, your sister/uncle/father also has HIV just like me and the virus works the same in all our bodies. As a family we are all helping to look after each other. We want you to feel part of that, and know that as a family, we are strong.

The best thing to do is to stay as close to the truth as possible, as this causes the least harm to children in the long term.

When this question comes up, the mother can also offer reassurance. The reason children ask these questions is because they feel anxious, so she can say something like:

I can see that you are a little worried about HIV and I can imagine that you might be worried about me or your sister. However, I don't want you to worry because you don't have HIV and you are not at risk of getting it from me or from anybody in the family. That is why I am teaching you today, because I want you to know about it and to learn about how I am staying healthy so that you don't have to worry.

It is important that the mother reminds the child that their job is to be a child and not to worry about adult things. For example, they could say:

Together we will fight this as a family. Because you now understand about it, you can also support your sister by making sure you have fun like you always do. My job is to make sure you all stay healthy. Your job is to be children, to play and to work hard at school.

This is a good place to move to the Playing Cards if the child has no further questions. So the mother can say something like:

Speaking of playing and having fun, I want to show you a fun game about staying healthy.

IMPORTANT LEARNING TIP:

It is important that mothers are prepared to deal with the issue of concordant and discordant siblings, if there are children in the family who have HIV. Children often bring this up, so it has to be dealt with. You should be aware of this from your discussions about the family situation in Session 1 – make sure that any HIV-positive children have been safely disclosed to first, before the mother begins telling HIV-negative children. Most importantly you will see that we recommend that the mother starts with the HIV-positive child. Making sure disclosure is safe for all children is important, so make sure you have checked on all of this before moving forward.

Illness and death related questions

Other common questions children ask are about illness and the threat of death. These are also the questions that mothers dread the most. Usually children will ask these questions after other HIV-related questions have come up and been addressed.

It is important to understand that children at this age are worried about the immediate future. The reason we have chosen to undertake

disclosure early is because we know that it is much easier for children to learn over time as they grow. We also know that it is very important that they learn about their parents' HIV status while their parent is still well. If they only learn about HIV after the parent is sick they can often feel much more threatened and scared. So you can reassure your mother that by doing early disclosure, this will be much easier and she can deliver some good news!

Children might ask something like:

- *Are you going to die?*

The mother should answer briefly. She should remember that children of this age do not want a long complicated story and, for now, they don't need to know every fact about death and dying. Instead they just need to be reassured that she is not sick now and she will not die today. She could say something like:

No, I am not sick at the moment, in fact my CD4 cells (spears) are still strong and I have not had to start medication yet. So you don't need to worry about me, I am living healthily with HIV.

No, I am not going to die soon, as long as I stay healthy/take my medication I can live a very long time with HIV. So you don't need to worry that I am going to die. Look at me, I am well, can you see that?

Yes, I have been sick from HIV before. Remember when I went to hospital? It was because my spears became weak and I had to start the medication, but can you see now that I am well and strong. That is the tablets that are making my shields strong.

Sometimes if parents have not started medication, their children will ask why not, and they may even be very anxious that the mother is going to get sick if she does not start treatment. The mother could respond:

I can see what you mean. You are worried that I must start taking medication soon so I don't become sick. But as you can see, my spears are still very strong so we can trust them to look after me for now. Every 6 months I go to clinic and they test me to see if the spears need help. When they do, I will start treatment, but not before.

Sometimes the child may ask about other family members who have died of HIV. For example, they might ask:

- *Did my father die of HIV?*
- *Did my father not take the tablets and that's why he died?*

Again, the mother should stay close to the truth, but also protect the child from difficult details which they do not need to know. For example she could say:

Yes, sometimes HIV gets the better of us. If it is sneaky and we don't realise we have it until its too late, or if we forget to take the medicine, then we can get sick and die. I don't want you to worry about me though. I am here and I know about my HIV and I know how to fight it. So, for now we are all fine.

Transmission related questions

Children may also ask about how HIV is transmitted. Again the mother should remember the rule of making sure that the answers are close to the truth, and offer the right amount of information. Children of this age do not need to know the technical details of transmission, so a simple explanation and deferring more in-depth conversation until they are older is an appropriate approach.

For example if a child asks:

- *Mum, how did you get HIV?*

The mother could answer:

When two people love each other and they are grown up, sometimes they sleep together very closely under the blanket, and at that time HIV may pass between them. I will teach you more about that when you are older, but for now I don't want you to worry about becoming infected, because you are safe from HIV. When you are older I will teach you more about how to prevent HIV infection, and when we visit the clinic you will also learn more about how to keep yourself healthy.

This would be a good time to move on to the health promotion messages. The mother could use the Playing Cards to reinforce several health messages:

I have a card game which teaches you about staying healthy. Can I show you? It might make you worry less about how I keep myself healthy.

The positive living messages on the Playing Cards can be used to supplement the HIV story-telling process. If the mother wants to offer any universal precaution messages, she could state something simple like:

HIV lives in the blood and in the fluids in the body. So when I have a wound or a cut, it is important for you not to touch it or to get blood on you. But with other things, like eating together, kissing or cuddling, or doing chores together, there is no risk.

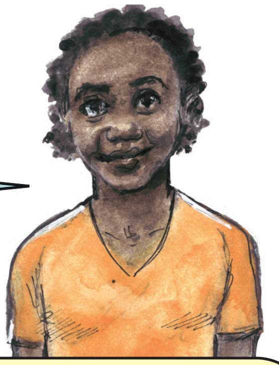

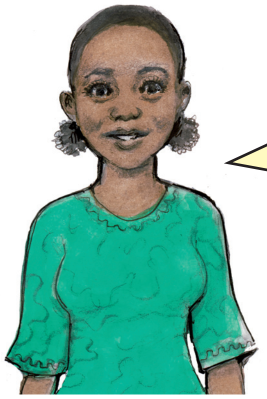

When the mother feels the child has asked enough, she can move on to the next step.
